# Back pain is also improved by lumbar disc herniation surgery

**DOI:** 10.1080/17453674.2020.1815981

**Published:** 2020-09-08

**Authors:** Niyaz Hareni, Fredrik Strömqvist, Björn Strömqvist, Freyr Gauti Sigmundsson, Björn E Rosengren, Magnus K Karlsson

**Affiliations:** a Department of Orthopaedics, Varberg Hospital, Varberg;; b Departments of Clinical Sciences and Orthopedics, Lund University, Skåne University Hospital, Malmö, Sweden;; c Department of Orthopedics, School of Medical Sciences, Faculty of Medicine and Health, Örebro University, Örebro, Sweden

## Abstract

Background and purpose — Indication for lumbar disc herniation (LDH) surgery is usually to relieve sciatica. We evaluated whether back pain also decreases after LDH surgery.

Patients and methods — In the Swedish register for spinal surgery (SweSpine) we identified 14,097 patients aged 20–64 years, with pre- and postoperative data, who in 2000–2016 had LDH surgery. We calculated 1-year improvement on numeric rating scale (rating 0–10) in back pain (N_back_) and leg pain (N_leg_) and by negative binomial regression relative risk (RR) for gaining improvement exceeding minimum clinically important difference (MCID).

Results — N_leg_ was preoperatively (mean [SD]) 6.7 (2.5) and N_back_ was 4.7 (2.9) (p < 0.001). Surgery reduced N_leg_ by mean 4.5 (95% CI 4.5–4.6) and N_back_ by 2.2 (CI 2.1–2.2). Mean reduction in N_leg_) was 67% and in N_back_ 47% (p < 0.001). Among patients with preoperative pain ≥ MCID (that is, patients with significant baseline pain and with a theoretical possibility to improve above MCID), the proportion who reached improvement ≥ MCID was 79% in N_leg_ and 60% in N_back_. RR for gaining improvement ≥ MCID in smokers compared with non-smokers was for N_leg_ 0.9 (CI 0.8–0.9) and ­N_back_ 0.9 (CI 0.8–0.9), and in patients with preoperative duration of back pain 0–3 months compared with > 24 months for N_leg_ 1.3 (CI 1.2–1.5) and for N_back_ 1.4 (CI 1.2–1.5).

Interpretation — LDH surgery improves leg pain more than back pain; nevertheless, 60% of the patients with significant back pain improved ≥ MCID. Smoking and long duration of pain is associated with inferior recovery in both N_leg_ and N_back_.

The most common indication for lumbar disc herniation (LDH) surgery is persistent sciatica that does not respond to nonoperative treatment (Blamoutier 2013). However, most patients who undergo LDH surgery also suffer from back pain (Hakkinen et al. 2003, Stromqvist et al. 2017), on a national level reported in 93% of patients having LDH surgery (Stromqvist et al. 2017). Decades ago, Mixter (1937) therefore argued that LDH extirpation should be accompanied by fusion to minimize postoperative back pain. Recent studies have opposed this view, showing that LDH surgery is not followed by increased back pain when only removing the hernia (Pearson et al. 2008, Owens et al. 2018), and in many cases even improvement of back pain seems sustainable over time.

Most studies that evaluate the outcome of LDH surgery focus on the relief from sciatica and improvement in patient-reported outcome measures (PROMs) (Weber 1983, Atlas et al. 2005, Peul et al. 2007, Weinstein et al. 2008, Lurie et al. 2014). A few studies have focused on back pain or included back pain in the evaluation (Kotilainen et al. 1993, Hakkinen et al. 2003, Toyone et al. 2004, Atlas et al. 2005, Pearson et al. 2008, Owens et al. 2018). While some of these infer that back pain is improved by the LDH surgery (Hakkinen et al. 2003, Toyone et al. 2004, Pearson et al. 2008, Owens et al. 2018) others report inconclusive results (Kotilainen et al. 1993, Atlas et al. 2005). There is a lack of consensus on the expected level of back pain reduction with LDH surgery.

It would also be of clinical interest to identify preoperative factors that are associated with favorable reduction of back pain following LDH surgery such as age, sex, smoking, preoperative health, and duration of pain (Nygaard et al. 2000, Jansson et al. 2005, Stromqvist et al. 2016, Wilson et al. 2016, Hareni et al. 2019).

We (i) evaluated whether back pain is reduced after LDH surgery and if so, to what extent compared with the reduction in leg pain and (ii) what proportion of patients gain improvement in back and leg pain exceeding minimum clinically important difference (MCID). The secondary aim was to identify factors associated with improvement in back pain exceeding MCID. 

## Patients and methods

Patient data was collected from the Swedish Spine Register (SweSpine), which is a patient register with prospectively collected data. The register covers 98% of all clinics performing lower back surgery in Sweden and has a completeness of 75% (www.swespine.se). In the register the patient reports preoperative anthropometric, lifestyle, and disease-related data such as age, gender, smoking habits (yes/no), numeric rating scale (NRS) for pain in the back (N_back_) and leg (N_leg_), duration of pain symptoms (categorized as no pain, pain 0–3 months, 3–12 months, 12–24 months, and > 24 months) and the PROM Short Form Health Survey 36 (SF-36) (rating from 0 to 100). The outcome after the operation is evaluated after 1 year by a similar questionnaire including current NRS pain level in back and leg and SF-36. The surgeon reports data concerning diagnosis, procedure, level of surgery, side of operation, and peri- and postoperative complications. SweSpine has previously been described in detail, including validation, with adequate results (Stromqvist et al. 2009).

We identified in SweSpine 19,815 patients aged 20–64 years during 2000–2016 with the diagnosis LDH and with baseline NRS back pain data. This age-span was chosen to include the typical LDH patient, albeit minimizing the risk of wrongful selection (that is, elderly patients with LDH diagnosis but also variable degree of spinal stenosis). The included patients had undergone open discectomy with or without microscope (87%), decompression with or without microscope (6%), various other types of surgeries (6%), or with type of surgery not reported (0.4%). 5,718/19,815 patients had not responded with postoperative NRS back pain data and were therefore excluded (Table 1). Patients included in this report had complete pre- and 1-year postoperative data for age, sex, and NRS back pain. All other included variables had above 96% response rates.

**Table 1.** Preoperative data in patients with both pre- and 1-year postoperative numeric rating scale (NRS) back pain data (n = 14,097) and in those with missing 1-year NRS back pain data (n = 5,718). Data are presented as numbers (n), proportions (%), or mean (standard deviation)

Factor	n	Patients with pre- and postoperative NRS back pain data	Patients with incomplete NRS back pain data
Mean age (SD)	14,097	43 (11)	41 (10)
Male sex (%)	14,097	55	61
Smokers (%)	13,975	19	25
Short Form-36 (SD)	13,930	47 (28)	45 (27)
NRS back pain (SD)	14,097	4.7 (2.9)	5.0 (2.9)
NRS leg pain (SD)	14,064	6.7 (2.5)	6.7 (2.5)
Duration of back pain (%)	13,191		
0–3 months		13	11
3–12 months		50	49
12–24 months		17	17
> 24 months		20	23
Duration of leg pain (%)	13,877		
0–3 months		17	15
3–12 months		54	53
12–24 months		15	16
> 24 months		14	15

### Statistics

IBM SPSS Statistics version 26 (IBM Corp, Armonk, NY, USA) was used for statistical analysis. Descriptive data are presented as numbers and means with standard deviations (SD) and inferential statistics as proportions (%) or means with 95% confidence intervals (CI). For group comparisons we used a paired Student’s t-test between means for continuous data and a chi-square test for categorical data. MCID was defined as an improvement by at least 2.5 units in NRS back pain and 3.5 units in NRS leg pain (Solberg et al. 2013). We used negative binomial regression to determine adjusted relative risk (RR) for preoperative factors that are associated with pain reduction ≥ MCID. We selected factors that in a previous publication have been found to be associated with general outcome in LDH surgery (Wilson et al. 2016). These variables included age, sex, smoking habit, quality of life (Short Form-36), and preoperative duration of pain. As binary dependent variable we dichotomized improvement in pain with improvement ≥ MCID being regarded as a successful and < MCID as an unsuccessful outcome. Furthermore, so as to be included in the binomial regression analyses, the patients had to have pain exceeding MCID at baseline (that is, having a hypothetical possibility to improve ≥ MCID). We regarded a p-value below 0.05 to indicate a statistically significant difference.

### Ethics, data sharing plan, funding, and potential conflicts of interest

The study was approved by the Lund regional ethical review board (Dnr 2017/158). Data sharing plan: the data is available from SweSpine upon request and approval by the registry board. No specific funding has been received for this study. No conflict of interest was declared. 

## ^Results^

N_leg_ was before surgery (mean [SD]) 6.7 (2.5) and N_back_ 4.7 (2.9) (p < 0.001), and 1 year after surgery 2.1 (2.7) and 2.5 (2.7) (p < 0.001) (Table 2). This corresponds to a reduction in N_leg_ by 4.5 (CI 4.5–4.6) and N_back_ by 2.2 (2.1–2.2). The relative N_leg_ reduction was 67% and relative N_back_ reduction 47% (Table 2). The proportion of patients who reached improvement ≥ MCID was 71% for leg pain and 43% for back pain (p < 0.001). When only including patients with pain ≥ MCID (3.5 for N_leg_ and 2.5 for N_back_), that is, patients with significant baseline pain and with hypothetical possibility to improve ≥ MCID, 79% of the patients improved ≥ MCID in leg pain and 60% in back pain (p < 0.001) (Table 2).

**Table 2.** Numeric rating scale 0–10 for back pain (N_back_) and leg pain (N_leg_) at baseline, 1 year after surgery, changes by surgery, and the proportion of patients with improvement ≥ MCID in pain (≥ 2.5 for N_back_ and ≥ 3.5 for N_leg_)

		Mean NRS (SD)		Changes in NRS (CI)		Proportion with improvement	
Factor	n	preoperatively	at 1 year	absolute	relative (%)	≥ MCID (%)	p-value
All patients							
N_back_	14,097	4.7 (2.9)	2.5 (2.7)	–2.2 (–2.1 to –2.2)	–47 (–45 to –47)	43	< 0.001
N_leg_	14,023	6.7 (2.5)	2.1 (2.7)	–4.5 (–4.5 to –4.6)	–67 (–67 to –69)	71	
Patients with preoperative back and/or leg pain ≥ MCID							
N_back_	9,975	6.2 (2.0)	3.0 (2.8)	–3.2 (–3.1 to –3.3)	–52 (–50 to –53)	60	< 0.001
N_leg_	12,268	7.4 (1.7)	2.2 (2.8)	–5.1 (–5.1 to –5.2)	–69 (–69 to –70)	79	

n — the number of patients who had answered this specific question;

SD —standard deviation; CI — 95% confidence intervals.

Smokers had, compared with non-smokers, RR of gaining improvement ≥ MCID in N_leg_ of 0.9 (CI 0.8–0.9) and in N_back_ of 0.9 (0.8–0.95). Older age (per year increment) had RR of gaining improvement ≥ MCID in N_leg_ of 0.995 (0.993–0.998) and in N_back_ of 1.00 (0.99–1.00). Duration of symptom 0–3 months had, compared to > 24 months, RR of gaining improvement ≥ MCID in N_leg_ 1.3 (1.2–1.5) and for N_back_ 1.4 (1.2–1.5), and 3–12 months compared with > 24 months for N_leg_ 1.2 (1.1–1.3) and for N_back_ 1.3 (1.2–1.4). Sex and quality of life by SF-36 was not associated with either outcome (Tables 3 and 4). 

**Table 3.** Negative binomial regression model presenting adjusted relative risk (RR) for different preoperative factors in respect of reaching improvement ≥ MCID in back pain in 9,674 patients with complete data

Factor	Mean RR (95% CI)
Age	1.0 (0.99–1.0)
Male (reference: female)	0.97 (0.91–1.0)
Smoker (reference: non-smoker)	0.9 (0.8–0.9) ^a^
Short Form-36	1.0 (0.99–1.0)
Duration of back pain (reference: > 24 months)	
0–3 months	1.4 (1.3–1.6) ^a^
3–12 months	1.3 (1.2–1.4) ^a^
12–24 months	1.1 (1.0–1.3) ^a^

To be included in this model, patients had to have N_back_ ≥ 2.5 at baseline. For continuous variables results are presented as RR per unit of change in the independent variable and for categorical variables compared with a reference category.

**^a^** Statistically significant.

**Table 4.** Negative binomial regression model presenting adjusted relative risk (RR) for different preoperative factors in respect of reaching improvement ≥ MCID in leg pain in 11,957 patients with complete data

Factor	Mean RR (95% CI)
Age	1.0 (1.0–1.0) ^a^
Male (reference: female)	0.95 (0.90–1.0)
Smoker (reference: non-smoker)	0.9 (0.8–0.95) ^a^
Short Form-36	1.0 (1.0–1.0)
Duration of back pain (reference: > 24 months)	
0–3 months	1.3 (1.2–1.5) ^a^
3–12 months	1.2 (1.1–1.3) ^a^
12–24 months	1.1 (0.99–1.2)

To be included in this model, patients had to have N_leg_ ≥ 3.5 at baseline. For continuous variables results are presented as RR per unit of change in the independent variable and for categorical variables compared with a reference category.

**^a^** Statistically significant.

## ^Discussion^

We found that both leg and back pain was reduced by LDH surgery, leg pain more than back pain, and that as many as 60% of the patients with back pain ≥ MCID reached back pain reduction equal to or above this level. Furthermore, non-smokers and patients with shorter duration of preoperative pain had a greater probability to reach leg and back pain reduction ≥ MCID. Younger age was associated only with leg pain reduction ≥ MCID. We also found that 79% of the patients with preoperative leg pain ≥ MCID had improvement defined as a clinically successful outcome, a success rate comparable to data in the literature (Solberg et al. 2013). By the same logic, the proportion of patients who achieved clinically successful back pain reduction was lower than the proportion with clinically successful leg pain reduction, but this was still considerable as back pain is not generally regarded as an indication for LDH surgery. The explanatory mechanism behind this back-pain reduction after LDH surgery is not clear (Peng et al. 2005, Yang et al. 2015).

Even though this study cannot conclude optimal timing for surgery, there was an association between shorter duration of symptoms (0–3 and 3–12 months compared with > 24months) and successful outcome. Peul et al. (2007) have found a similar 1-year outcome for those operated on early (6–12 weeks’ duration) and those who waited another 6 months.

We highlight that our study design could draw inferences only as regards associations. Our results do not motivate LDH surgery on the basis of back pain. We can only use these data to inform patients scheduled for LDH surgery that they have a probability of 60% of having a reduction of clinical significance if they have back pain ≥ MCID. Our view is supported by another registry study that included 2,262 patients, which reported that patients with baseline back pain NRS ≥ 5 (out of maximum 10), also had significant improvement in the back pain by discectomy (Owens et al. 2018). However, that study did not evaluate the proportion of patients who reached the MCID level of pain reduction. Further studies should examine whether it is possible to identify sub-groups of patients that will specifically benefit from back-pain reduction by discectomy.

Strengths in our study include a large study population, prospectively collected data, and reports of outcome on a national level that identify the results in the general health care system rather than in highly specialized spinal units. Another strength is the absence of exclusion criteria, rendering the actual outcome within general health care, in which patients with comorbidities and relative contraindications for surgery are included. This should be compared with studies that show what it is possible to achieve in selected defined patient cohorts in specialized units with highly trained surgeons (Kotilainen et al. 1993, Staartjes et al. 2019). Limitations include those unavoidable in registry-based studies, such as incomplete pre- and postoperative data collection. These limitations have not, however, biased the outcome effects (Solberg et al. 2011). Another weakness is the inability to adjust for all possible confounders, such as radiological evaluations to assess findings that have been reported in the literature to be associated with back pain (Yang et al. 2015) and data on more possible confounders to include in our model.

In conclusion, both leg and back pain improve after LDH surgery, leg pain more than back pain. In patients with preoperative pain ≥ MCID, leg pain level was reduced at this level or above in 79% of the patients and back pain in 60%. The preoperative factors that in our model were associated with back pain reduction ≥ MCID by LDH surgery were virtually the same as those associated with leg pain reduction ≥ MCID. Our results improve the ability to provide accurate preoperative information as regards the probability of reaching clinically significant reduction in back and leg pain by LDH surgery. 
